# Dendritic Cell Vaccination in Conjunction with a TLR Agonist Polarizes Interferon Immune Responses in Malignant Glioma Patients

**DOI:** 10.21203/rs.3.rs-3287211/v1

**Published:** 2023-09-12

**Authors:** Richard G. Everson, Willy Hugo, Lu Sun, Joseph Antonios, Alexander Lee, Lizhong Ding, Melissa Bu, Sarah Khattab, Carolina Chavez, Emma Billingslea-Yoon, Andres Salazar, Benjamin M. Ellingson, Timothy F. Cloughesy, Linda M. Liau, Robert M. Prins

**Affiliations:** 1 Department of Neurosurgery, David Geffen School of Medicine at UCLA, University of California Los Angeles, Los Angeles, California, 90095, U.S.A.; 2 Jonsson Comprehensive Cancer Center, David Geffen School of Medicine at UCLA, University of California Los Angeles, Los Angeles, California, 90095, U.S.A.; 3 Department of Medicine, Division of Dermatology, David Geffen School of Medicine at UCLA, University of California Los Angeles, Los Angeles, California, 90095, U.S.A.; 4 Parker Institute for Cancer Immunotherapy, David Geffen School of Medicine at UCLA, University of California Los Angeles, Los Angeles, California, 90095, U.S.A.; 5 Department of Molecular and Medical Pharmacology , David Geffen School of Medicine at UCLA, University of California Los Angeles, Los Angeles, California, 90095, U.S.A.; 6 Oncovir, Inc., Winchester, VA; 7 Department of Radiological Sciences, David Geffen School of Medicine at UCLA, University of California Los Angeles, Los Angeles, California, 90095, U.S.A.; 8 Department of Neurology/Neuro-Oncology, David Geffen School of Medicine at UCLA, University of California Los Angeles, Los Angeles, California, 90095, U.S.A.; 9 Richard Everson and Willy Hugo contributed equally to this work as first authors

**Keywords:** brain cancer, immunotherapy, glioma, TLR agonist

## Abstract

Autologous tumor lysate-pulsed dendritic cell (ATL-DC) vaccination is a promising immunotherapy for patients with high grade gliomas, but responses have not been demonstrated in all patients. To determine the most effective combination of autologous tumor lysate-pulsed DC vaccination, with or without the adjuvant toll-like receptor (TLR) agonists poly-ICLC or resiquimod, we conducted a Phase 2 clinical trial in 23 patients with newly diagnosed or recurrent WHO Grade III-IV malignant gliomas. We then performed deep, high-dimensional immune profiling of these patients to better understand how TLR agonists may influence the systemic immune responses induced by ATL-DC vaccination. Bulk RNAseq data demonstrated highly significant upregulation of type 1 and type 2 interferon gene expression selectively in patients who received adjuvant a TLR agonist together with ATL-DC. CyTOF analysis of patient peripheral blood mononuclear cells (PBMCs) showed increased expression of PD-1 on CD4+ T-cells, decreases in CD38 and CD39 on CD8+ T cells and elevated proportion of monocytes after ATL-DC + TLR agonist administration. In addition, scRNA-seq demonstrated a higher expression fold change of IFN-induced genes with poly-ICLC treatment in both peripheral blood monocytes and T lymphocytes. Patients who had higher expression of interferon response genes lived significantly longer and had longer time to progression compared to those with lower expression. The results suggest that ATL-DC in conjunction with adjuvant poly-ICLC induces a polarized interferon response in circulating monocytes and specific activation of a CD8+ T cell population, which may represent an important blood biomarker for immunotherapy in this patient population.

**TRIAL REGISTRATION:**

ClinicalTrials.gov Identifier: NCT01204684

## INTRODUCTION

There have been significant advances in our genetic and immunologic understanding of primary brain tumors, such as malignant gliomas. Yet, it has still proven difficult to improve long-term outcomes in patients using standard of care therapies ^1^. We and others have demonstrated that autologous tumor lysate (ATL) dendritic cell (DC) vaccination can induce local and systemic anti-tumor immune responses in malignant glioma patients, and clinical trials have suggested that this may extend survival in this deadly condition^2–6^. However, variable response rates in cancer immunotherapy trials have prompted the search for potential strategies to enhance the immune effects of dendritic cell vaccines. In particular, agonists of a family of pattern-recognition receptors (PRR) called Toll-like receptors (TLR)^7–10^, which appear capable of activating of antigen-presenting cells (i.e., dendritic cells), enhancing T-cell priming, and decreasing myeloid-derived suppressor cells (MDSC), are strong candidates for use in combination with ATL-DC vaccination to potentially enhance the anti-tumor immune response.^10,11^

TLR3 is an intracellular PRR that recognizes double-stranded RNA (dsRNA), usually associated with viral infection, and induces high levels of IFN-α/β and pro-inflammatory cytokines when activated. TLR-3 is predominantly expressed by macrophages, plasmacytoid DC and myeloid DC ^12,13^, but also by microglial cells ^14,15^. It has also been shown that astrocytes^16–18^ and malignant gliomas^19^ also respond similarly to TLR3-induced signaling. Polyinosinic acid-polycytidylic acid stabilized with polylysine (poly-ICLC) is a multi-dimensional synthetic dsRNA analogue and viral mimic that signals via TLR3, MDA5 and other dsRNA-dependent PRR signaling, induces type I-II IFNs^20,21^, promotes the infiltration of effector T cells in pre-clinical glioma models^22^, and upregulates genes associated with chemokine activity, T-cell activation, and antigen presentation^23^. Poly-ICLC has been tested as a single-agent therapeutic for multiple malignancies^24^, including malignant glioma patients ^25^, in whom it has demonstrated adequate safety, but limited survival benefit in combination with standard therapies^26^.

Similarly, TLR7 and TLR8 are other intracellular PRRs that recognize single-stranded RNA (ssRNA), which subsequently induces proinflammatory cytokines, chemokines, and type I interferons (IFNs) ^27^. In pre-clinical work, we previously demonstrated that DC injected into imiquimod (TLR7 agonist)-pre-treated sites acquired lymph node migratory capacity and enhanced T-cell priming ^28^. Our early phase clinical trials demonstrated that DC vaccination with adjuvant topical imiquimod, a TLR-7 agonist, was feasible and safe in glioblastoma patients ^3^. Resiquimod is a newer imidazoquinoline agonist that shows enhanced transdermal delivery, activates TLR7/8 to enhance T-cell responses and TH1-type cytokine secretion by DC ^29–32^, and may have greater potency as an immune modulator.

In this study, we report the long-term results of 23 malignant glioma patients enrolled in a phase II randomized clinical trial designed to compare the safety, immune responses, and potential efficacy of ATL-DC vaccination combined with placebo, poly-ICLC, or resiquimod. Post-hoc analysis using cytometry by time-of-flight (CyTOF) and bulk and single-cell RNA sequencing (scRNAseq) technologies were used to detect the cellular and molecular immune signatures from peripheral blood mononuclear cells (PBMCs) pre- and post-treatment.

## RESULTS

### Patient Characteristics and Safety

**Figure 1.**
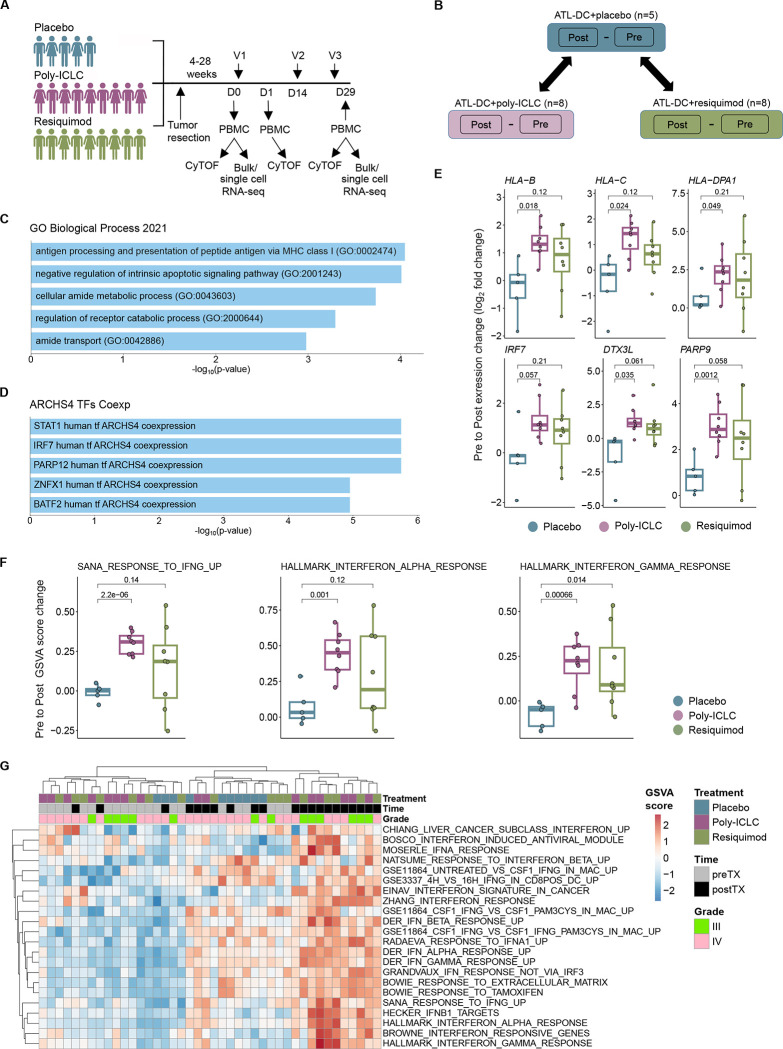
Combination of ATL-DC vaccine and TLR agonists results in a robust interferon pathway activation in the patient PBMCs. **A,** Timeline of PBMC acquisition and analysis using CyTOF and/or RNAseq. V = vaccine, D = Day. **B,** Schematic of differential gene expression analysis performed on pre-treatment and post-treatment PBMCs of indicated treatment groups. Differentially expressed genes (DEGs) in TLR agonist-treated groups are compared against their changes in the placebo group to identify DEGs specific to the TLR-agonist groups. **C, D,** Enriched gene set terms in Gene Ontology Biological Process (C) or ARCHS4 TF Coexp datasets that significantly overlap with the union of DEGs from ATL-DC + poly-ICLC and ATL-DC + resiquimod groups (P values, FDR-adjusted, two-sided fisher exact test). **E,** Differential gene expression (pre vs. post-treatment fold change, in log2) of representative antigen presentation and IFN related genes across treatment groups (P values, two-sided two-sided Welch t test). **F,** Gene set enrichment score differences (pre vs. post-treatment, delta GSVA score) of representative IFN related genesets across treatment groups (P values, two-sided Welch t test). **G,** Heatmap of single-sample, gene set enrichment scores (GSVA) of type I and type II interferon genesets in pre-treatment, ATL-DC + placebo, ATL-DC+poly-ICLC and ATL-DC+resiquimod samples.

A total of 23 patients with WHO grade III or IV glioma were enrolled and randomized between September 2010 and August 2014. All patients received ATL-DC vaccination. Nine patients were randomized into the adjuvant TLR-7/8 agonist (resiquimod, 3M) group, nine into the adjuvant TLR-3 agonist (poly-ICLC, Oncovir) group, and five received adjuvant placebo (Figure 1A, Supplementary Figure 1). All patients were followed for survival, imaging changes, as well as high dimensional, in-depth systemic immune monitoring. Baseline patient characteristics are presented and segregated by treatment group in Table 1 (see also Supplementary Table 1). The median age was 46.6 (S.D. 11.9) years and 57% of the enrolled patients were male.65% (n=15) had diagnoses of IDH wild type glioblastoma (WHO Grade IV), while 35% (n=8) of the patients had a diagnosis of IDH mutant malignant glioma (WHO Grade III). 52% (n=12) of patients were treated following recurrence, while 48% (n=11) were treated in the newly diagnosed setting. All patients were treated following surgical resection and standard of care treatment. The molecular characteristics of the patient tumors are outlined in Table 1. Overall, MGMT methylation was seen in 35% (n=8), IDH mutations were observed in 35% (n=8, all grade III), and EGFR amplification was seen in 44% (n=10, all glioblastoma) of patients, consistent with the heterogenous population of malignant glioma patients. There were no statistically significant differences in age, sex, Karnofsky performance status, MGMT methylation status, pre- or post-surgery enhancing tumor volume, nor steroid administration at enrollment. No statistically significant differences were observed between the molecular characteristics, although the number of patients in each treatment group was small.

Overall, the addition of a TLR agonist induced only Grade 1–2 treatment-related adverse events (TRAEs), and all adverse events reported resolved without further treatment or hospitalization (Table 2). The most common TRAEs were rash (39%), fever (35%), and fatigue (26%; see Table 2), and were more common in patients treated with resiquimod and poly-ICLC. Other observed adverse events were not uncommon in the setting of post-operative central nervous system (CNS) tumor treatment. Additionally, 88.9% of patients who received resiquimod reported a temporary localized, cutaneous rash that resolved without further treatment. However, no serious adverse events (Grade 3–4) attributable to the treatment were observed. As such, the addition of a TLR agonist to ATL-DC vaccination in malignant glioma patients was found to be safe and tolerable.

### Adjuvant TLR agonist treatment induces systemic expression of type I and type II interferon downstream genes.

The primary scientific endpoint of this clinical trial was to evaluate the systemic immune response changes induced by ATL-DC vaccination with and without TLR agonist administration. As such, we collected PBMCs at baseline (pre-treatment), one day after the vaccination (on treatment), and then following the completion of the treatment cycle (post-treatment) of each patient (Figure 1A). We aimed to understand how the adjuvant administration of TLR agonists modified the immune response in comparison with ATL-DC vaccination alone (placebo control). We first performed paired bulk RNA-seq on patient-matched, pre-treatment and post-treatment PBMC samples. For each gene, we computed the difference between its expression in the pre-and post- samples of patients in each treatment group: ATL-DC+placebo (n=5 pairs); ATL-DC+poly-ICLC (n=8 pairs); ATL-DC+resiquimod (n=8 pairs); for brevity, we refer to them as placebo, poly-ICLC and resiquimod, respectively. To identify expression changes specific to the TLR agonist groups, we identified genes whose average upregulation in the TLR agonist pairs (poly-ICLC or resiquimod) were at least two-fold higher than the placebo pairs (Figure 1B, Supplementary Table 2A, see Methods).

Genes upregulated in the TLR agonist groups were involved in antigen processing and were enriched with known interferon stimulated genes (ISGs) (Figure 1C–E, Supplementary Table 2B–C). This observation was also confirmed by per-sample gene set enrichment analysis, where the TLR agonist-treated groups displayed higher enrichment of type I and II interferon downstream gene sets compared to ATL-DC/placebo (Figure 1F, Supplementary Table 2D–E). PBMC samples with higher absolute enrichment scores of interferon gene sets were dominated by post-treatment samples from both grade III and IV glioma patients in the TLR-agonist treated groups (Figure 1G). We noted that the resiquimod group had a more heterogenous response, which resulted in a lower degree of statistical significance compared to that of poly-ICLC group. Nonetheless, the two TLR agonist-treated groups showed a largely similar trend in treatment-induced gene expression changes, which included a measurable increase in the expression of ISGs in the peripheral blood of malignant glioma patients.

### TLR agonist treatment induces systemic T cell activation, monocyte proliferation and interferon responses in myeloid and lymphoid populations.

**Figure 2.**
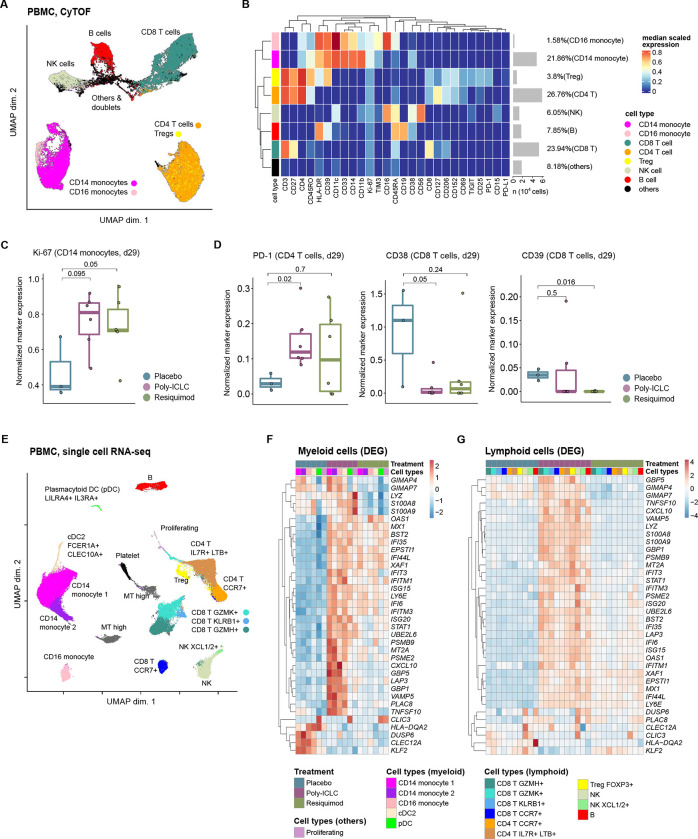
Single cell analysis reveals activation of systemic T cells and monocytes as a part of interferon pathway activation in all myeloid and lymphoid populations. **A,** A UMAP projection of the pre- and post-treatment PBMC sample pairs from twenty patients (placebo, n=4 pairs; poly-ICLC, n=9 pairs; resiquimod, n=7 pairs). Clustering was performed with a random sampling of 5,000 cells from each patient. **B,** Heatmap of normalized expression of all 27 cell markers within cell populations identified in the patient PBMCs. **C, D,** Normalized expression of indicated markers in monocyte (C), or T cell populations (D) within the PBMC samples of patients from indicated treatment groups. P values, two-sided Wilcoxon rank sum test. **E,** UMAP projection of the PBMC-derived single cells (n=99,590). Immune subset associated with each cluster is inferred based on the cluster’s differentially expressed transcripts. Canonical markers of known immune subsets are shown. **F, G,** Heatmaps showing the union of recurrent DEGs computed between ATL-DC treated samples (combined with placebo, resiquimod or poly-ICLC) and pre-treatment samples in the myeloid populations (F) or lymphocyte populations (G). Shown in the heatmaps are the log fold change values of the DEGs in each cell population grouped by their treatment groups.

We performed CyTOF on PBMC timepoints with a 27-marker heavy metal antibody-conjugated panel for 20 of the 23 patients (placebo, n=4 pairs; poly-ICLC, n=9 pairs; resiquimod, n=7 pairs; see Supplementary Table 3A, B). The panel was selected to be able to broadly characterize different immune cell types, activation/effector, memory and exhaustion phenotypes, with a bias towards T-cell relevant markers. The different immune cell type populations were visualized by the uniform manifold approximation and projection (UMAP) method (Figure 2A), which we broadly assigned to seven different major immune populations based off the normalized heatmap marker expression (Figure 2B).

After 3 cycles of treatment, the post-treatment samples of patients in the TLR agonist groups showed a significant increase in the proportion of proliferating Ki67+ CD14+ classical monocytes (Figure 2C, Supplementary Table 3C). Such findings were supported by the increased monocyte fraction and *CD14* transcript expression after ATL-DC+TLR agonist-treated samples (Supplementary Figure 2A, B, Supplementary Table 3D). ATL-DC+TLR agonist treatment induced PD-1 expression in CD4 T cell population and increased the T-cell normalized expression of *PDCD1* (the transcript that encodes PD-1 protein) (Figure 2D, Supplementary Figure 2C). Moreover, expression of markers associated with irreversible T cell exhaustion, such as CD38 and CD39 ^33,34^, were also reduced after ATL-DC+TLR agonist treatment (Figure 2D, Supplementary Figure 2D). Increased expression of PD-1 and decreased expression of CD38 and CD39 suggest the ATL-DC+TLR agonist combination therapy can improve systemic T cell activity and cellular fitness in the patient.

To delineate the changes induced by ATL-DC and TLR agonist treatment in discrete peripheral blood immune cell subsets, we performed single cell RNA-seq on selected patients at baseline and then following the completion of therapy. We analyzed two representative sample pairs from each cohort (placebo, poly-ICLC, and resiquimod) (Supplementary Table 3E). We identified a total of twelve clusters from the total PBMC immune cell population and annotated these clusters based on differentially expressed gene markers in each cluster. From the initial clustering, we were able to identify multiple populations of CD4^+^ and CD8^+^ T cells, two populations of NK cells, three monocytic cell populations, B cell, and dendritic cells (type 2 conventional dendritic cells (cDC2) and plasmacytoid dendritic cells (pDCs), in accordance with the previous characterization of these cell types in peripheral blood (Figure 2D and Supplementary Figure 2E, F).

Differential gene expression analysis across the different lymphoid and myeloid populations revealed concordant upregulation of known ISGs (e.g. *IFI6/35/44L, ISG15/20*, *IFIT3*, *IFITM1/3*, *GBP1/5*, *MX1, STAT1 and CXCL10*) and antigen presentation related proteasomes (*PSMB9* and *PSME2*) in both TLR agonist sample pairs. The induction was weaker in the paired PBMC samples obtained from the resiquimod group compared to the poly-ICLC group (Figure 2F, G).

Thus, our combination of high dimensional proteomics, bulk and single cell RNAseq demonstrates how adjuvant TLR administration in conjunction with ATL-DC reproducibly increases the proportion of canonical CD14+ monocytes within the systemic blood circulation. This TLR agonist administration was also associated with enhanced T cell activity, coupled with decreased expression of CD38 and CD39 and their downstream T cell-suppressive adenosine pathway ^33–35^ . ATL-DC+TLR agonist-driven induction of ISGs across lymphoid and myeloid populations identified in our scRNAseq analysis corroborated our bulk transcriptomic analysis. Given the consistent systemic changes observed with TLR agonist administration, we wondered if there were survival differences between these patient populations.

### Long-term clinical outcomes of malignant glioma patients treated with ATL-DC vaccination plus adjuvant TLR agonists.

**Figure 3.**
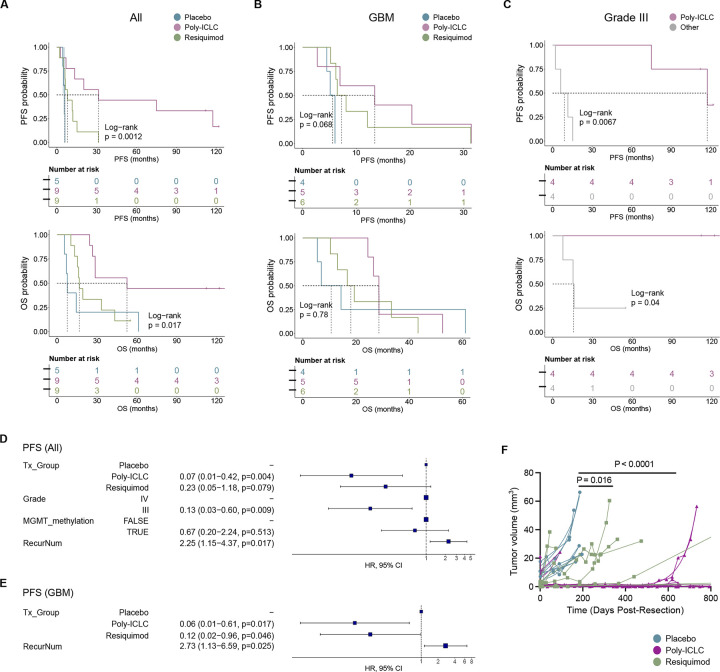
Combined ATL-DC vaccine and TLR agonist treatment show trends of improved tumor control and patient survival. **A, B, C,** Progression-free survival (PFS, top) and overall survival (OS, bottom) of all patients (A), patient subset with GBM (B), or grade III glioma (C) in indicated treatment groups. P values, log-rank test. **D, E,** Multivariate Cox proportional hazards analysis assessing the hazard ratios of tumor progression in TLR agonist treatment groups against placebo in all patients (D) or GBM subset after adjusting for other clinical covariates (Tx_Group=treatment group, RecurNum=number of recurrences prior to ATL-DC treatment). **F,** MR-computed volumes of post-treatment, recurrent tumors in indicated treatment groups. P values, unpaired, two-sided Wilcoxon rank sum test.

Median follow-up of patients treated on this clinical trial was 2.2 years after surgery, although the long-term survivors have now been followed for over 10 years. Median progression-free survival (PFS) was 8.1 months; and median overall survival (OS) was 26.6 months. Although this clinical trial was not designed to be statistically powered to detect changes in survival between the treatment groups, we nonetheless noted statistically significant differences in median survival between the treatments groups for both OS (placebo: 7.7 months, poly-ICLC: 52.5 months, and resiquimod: 16.7 months; log-rank P=0.017) and PFS (placebo: 5.5 months, poly-ICLC: 31.4 months and resiquimod: 8.1 months; log-rank P=0.0012) (Figure 3A). Because the trial included patients with both grade III and IV tumors, we stratified our analysis based on tumor grade. When we analyzed only the grade IV (GBM) patients, we observed a trend towards improved PFS (log-rank P=0.068) and OS (P not significant) (Figure 3B). Interestingly, for the IDH mutant/Grade III cohort, all four patients that received ATL-DC + poly-ICLC treatment are still alive at the data cutoff date (three of the patients have survival > 120 months and one > 112 months), and they have significantly longer OS and PFS compared to the other grade III patients who received ATL-DC + resiquimod or ATL-DC alone (Figure 3C).

We performed multivariate Cox proportional hazard (PH) analysis, adjusting for clinical variables that are significantly correlated with OS or PFS as a single variable (tumor grade, MGMT methylation status, and number of recurrences). Our analysis confirmed that patients in either the poly-ICLC or resiquimod treatment group had a lower risk of progression that was independent of grade, MGMT methylation, and number of recurrences (Figure 3D). Risk of death was significantly lower in the poly-ICLC group, while the resiquimod group showed a similar trend that was not statistically significant (Supplementary Figure 3A). In the GBM patient subset, TLR agonist treatment also significantly lowered risk of recurrence, but not risk of death (Figure 3E, Supplementary Figure 3B).

To determine whether this treatment directly impacted tumor volume, MR imaging was performed, and contrast-enhancing tumor volume was quantified over time. We noted that the rate of tumor volume increase over time in the ATL-DC/placebo treatment cohort was higher than in the ATL-DC/resiquimod treatment (p=0.022) and the ATL-DC/poly-ICLC treatment groups (P < 0.001; Figure 3F). Interestingly, we observed an increased T2/FLAIR MRI signal after completion of the vaccine series two of the four long-term survivors who received ATL-DC/poly-ICLC (Supplementary Figure 3C–D), but such findings are potentially confounded by prior radiation therapy, and thus we cannot ascribe such changes solely to the vaccine/TLR agonist intervention. However, this increased post-vaccination T2/FLAIR on MRI was not seen in patients who did not receive poly-ICLC (not shown).

### Interferon activation score in the peripheral blood immune cells is a significant predictor of survival after ATL-DC therapy.

**Figure 4.**
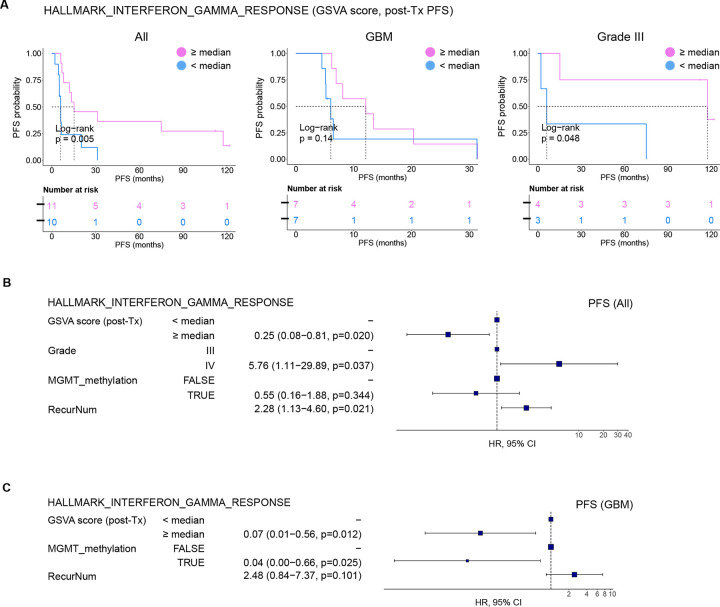
IFN pathway activation is a positive predictor of survival after ATL-DC vaccine and TLR agonist therapy. **A,** Kaplan-Meier progression-free survival curves of all patients (left), GBM (center), and Grade III glioma subsets (right) stratified by their HALLMARK_INTERFERON_GAMMA_RESPONSE GSVA scores in their post-treatment PBMCs. P values, log-rank test. **B, C,** Multivariate Cox proportional hazards analysis assessing hazard ratios of tumor progression in patients with high HALLMARK_INTERFERON_GAMMA_RESPONSE GSVA score of in all patients (B) or GBM subset (C) after adjusting for other clinical covariates.

Finally, we asked if the magnitude of interferon pathway induction by the adjuvant TLR agonist treatment directly correlated with OS or PFS. This could allow for the use of an interferon activity score as a biomarker for productive anti-tumor immune responses following ATL-DC immunotherapy. To this end, we stratified the patients by the median GSVA score of the “HALLMARK INTERFERON GAMMA RESPONSE” gene set in their post-treatment PBMC samples. We confirmed that patients whose post-treatment samples displayed higher interferon gene set scores (≥ median) have longer OS and PFS than those with lower scores (Figure 4A, Supplementary Figure 4A). Separate analyses on the grade IV (GBM) and grade III glioma patients showed a concordant trend but with a lower degree of statistical significance; this was likely caused by the small sample sizes. Notably, multivariate Cox PH analysis confirmed that the interferon gene set score is a significant predictor of tumor recurrence (Figure 4B, C) and death (Supplementary Figure 4B). To ensure that the correlation is not specific to this single gene set, we confirmed that the gene set scores of other interferon gene sets after ATL-DC treatment are also positively correlated with the patients OS and PFS (Supplementary Table 4A, B).

Taken together, these data suggest that the addition of TLR agonists to ATL-DC vaccination shifts towards an interferon-induced immune response in both lymphoid and myeloid cells. The magnitude of this induction may be correlated with the systemic immune response and clinical outcome.

## DISCUSSION

We report herein that ATL-DC vaccination with adjuvant poly-ICLC or resiquimod is overall safe and well-tolerated in patients with malignant glioma. To achieve the primary immunological endpoints of this study, we utilized high-dimensional single-cell analysis to understand the systemic proteomic and transcriptomic changes induced by agonists of TLRs and other PRR in order to rationally determine the optimal therapeutic combination.

Our study is the first high dimensional single-cell analysis done in a clinical trial for malignant glioma patients treated with dendritic cell vaccination and TLR agonists. Although our study was not designed to examine what happens in the tumor microenvironment, our results indicate that we are able to sensitively detect systemic changes in the blood after intradermal autologous dendritic cell vaccination with and without TLR agonists. Adjuvant TLR agonist treatment promotes the expression of IFNα/β and IFNγ-induced genes on the peripheral lymphoid and myeloid cells, and GSEA further confirmed increased expression of the IFNα and IFNγ downstream genes, including IFNα/β-induced proteins *ISG15* and *STAT1*. *ISG15* stimulates IFNγ from lymphocytes ^36^ and negatively regulates IFNα/β signaling ^37^, and type I IFN maintenance of *STAT1* expression induces IFNγ signaling ^38^. Other genes that were significantly upregulated by TLR agonist treatment include PARP9-DTX3L, and this heterodimer is also known to amplify interferon signalling.^39^ Our results support the conclusion that DC vaccination with poly-ICLC induces Type I and Type II IFN responses more effectively than with adjuvant resiquimod or a dendritic cell vaccine alone. Similar to our results, additional studies have identified poly-ICLC as the most effective TLR/PRR agonist when compared with others^40,41^. The downstream effect of this signaling in the lymphoid compartment appears to be increased T-cell activity, as well as decreased T cell exhaustion phenotype. Together, these effects may enhance of the activity of tumor antigen specific T cells generated from an active vaccine.

It is also important to recognize that, in contrast to resiquimod and even plain poly-IC, poly-ICLC signals through various PRRs in addition to TLR3, consistent with its role as an authentic viral mimic. The poly-lysine stabilizer also functions as a transfection agent. Specifically, poly-lysine bursts the endosome through a proton sponge effect and releases the dsRNA into the cytoplasm, where it then preferentially activates MDA5, OAS, PKR and other cytoplasmic dsRNA dependent systems ^21^. Among the actions generated putatively through MDA5 are a further increase in Type 1 IFN, depression of MDSC, expansion of CD8 T cell populations through IL-15, CD8 targeting and infiltration of tumor through CXCL10, and a direct Type 1 IFN-dependent effect on tumor endothelium through VCAM-1 ^42^. These effects are best seen with systemic (intramuscular or intravenous) rather than local (subcutaneous) administration, as we have done in the current study. Such adjuvant responses induced by poly ICLC may play a role in the longer-term maintenance of the immune responses generated by ATL-DC vaccination, but further studies are required to verify these findings.

While malignant gliomas are usually conceived of as a locoregional disease with essentially no capacity to spread outside the central nervous system, there has been a growing understanding of the role that systemic tissues play in priming, developing and/or suppressing an immune response in the brain. The catalog of known pervasive systemic immune deficits in glioblastoma patients is continually growing ^43^. The failure of immune checkpoint inhibitor therapy in malignant gliomas has led many to conclude that immune cells in the tumor microenvironment of cancers unresponsive to these checkpoint inhibitors may exist in an irreversible, terminally exhausted state^44,45^. The generation of *de novo* tumor antigen-specific immune responses in the periphery that lead to new T-cell infiltration into the tumor microenvironment may be required to overcome this barrier^46^. Dendritic cell vaccines are a robust example of an agent capable of mediating the initiation of such a T-cell response.

We were able to detect an immunosuppressive phenotype in the myeloid cells of the placebo treatment cohort. Monocytes and DCs after ATL-DC/placebo vaccination showed upregulation of *CLEC12A,* which is a recently characterized inhibitory pattern recognition receptor expressed selectively on myeloid cells. This C-type lectin receptor is known to be downregulated following activation^47^ and thought to control noninfectious inflammation ^48^. Gene expression of CLEC12A was absent in the ATL-DC/poly-ICLC group and only slightly increased in the ATL-DC/resiquimod group. The fraction of monocytes in the systemic circulation is known to be an important biomarker for the response to PD-1 checkpoint blockade immunotherapy ^49^. In conjunction with our other findings that the TLR agonists induced a higher fraction of CD14+ classical monocytes in the blood, such data suggest that the combination of ATL-DC+TLR agonist with immune checkpoint blockade may be a rational choice. In fact, we have now initiated a phase I trial combining ATL-DC+Poly-ICLC with pembrolizumab in recurrent glioblastoma patients (NCT04201873).

While encouraging, our clinical findings must be interpreted with caution. Even though this was a randomized clinical trial, the small number of patients enrolled likely contributed to an imbalance in patient selection between the treatment groups. The patients randomized to the resiquimod group and poly ICLC group were approximately consistent, but the patients in the ATL-DC + placebo had more unfavorable clinical characteristics. We found that ATL-DC vaccinated patients randomized to receive adjuvant TLR agonists demonstrated a statistically significant extended overall time to tumor progression and slower rates of tumor growth, compared with those who received adjuvant placebo. The poly-ICLC group was further associated with a statistically significant increase in overall median survival. Some of the grade III gliomas treated with ATL-DC/poly-ICLC exhibited unique T2/FLAIR changes on brain MRI scans following DC vaccination, but such findings were confounded by previous radiation therapy, even though such changes were not seen in the other patients. The significance of these imaging findings is not clear and needs to be replicated.

In conclusion, we demonstrate that autologous dendritic cell vaccination plus TLR agonists in patients with malignant gliomas generates a systemic interferon activation signature in the peripheral blood that is correlated with overall survival. Although this was a randomized study, it was powered for immune biomarker analysis, not for survival. As such, the clinical efficacy outcomes should be interpreted with caution. Given the noted long-term survival with the adjuvant use of poly-ICLC with DC vaccination, particularly in the grade III cohort of patients, further clinical trials that incorporate these combinations of immunotherapeutic agents are warranted.

## METHODS

### Study design.

This was a single-center, randomized, open-label multi-arm phase II clinical trial. The study protocols were approved by independent ethics committees and institution review boards as required. Patients were recruited and completed treatment between 2010 and 2014, with survival follow-up until the present date. All patients gave written informed consent before enrollment.

Twenty-three patients with high-grade WHO Grade III or IV gliomas were enrolled in this protocol. To be eligible for the primary cohort, patients had to be >18 years and have newly diagnosed or recurrent WHO Grade III or IV malignant glioma, as determined through central pathology review. For all patients, a Karnofsky Performance Score (KPS) of ≥ 60, adequate bone marrow, liver, and renal function, life expectancy of ≥ 8 weeks, no other prior malignancy within the last 5 years, no active viral infections, and sufficient resected tumor material to produce the autologous vaccine were required. All newly diagnosed patients underwent surgical resection followed by radiation and chemotherapy with temozolomide for 6 weeks, per standard of care. Patients in the recurrent setting proceeded to trial treatment, after recovery from surgery. All patients were scheduled to receive ATL-DC. Patients were then randomized to receive either placebo, resiquimod (topical 0.2%, 3M), or poly-ICLC (20 mcg/kg i.m., Oncovir) as an adjuvant to the DC vaccine. Patients underwent leukapheresis to obtain adequate numbers of PBMC for DC generation. For the study treatment, we processed the resected tumor tissue into a lysate, then prepared and cryopreserved the autologous DCs as we previously described^2,3^. Patients were then treated with three intradermal injections of autologous tumor lysate-pulsed DC plus adjuvant TLRs/placebo on days 0, 14, and 28. Follow-up for patients was conducted at the study site for vital signs, KPS, hematology and serum chemistries, as well as neurological and physical examinations.

### Clinical assessments.

Safety was assessed on the basis of occurrence of adverse events, which were categorized according to the NCI Common Toxicity Criteria for Adverse Events v. 4.0. Safety assessments were performed on the day of vaccination and 1 week after each vaccination during the treatment phase, and every 2 months thereafter until tumor progression or death.

Anatomic MR images were acquired prior to DC + adjuvant treatment and at 2-month intervals for all patients using the standardized brain tumor imaging protocol (BTIP)^50^, including three dimensional pre- and post-contrast T1-weighted images at 1–1.5mm isotropic resolution, two-dimensional T2-weighted and T2-weighted fluid attenuated inversion recovery (FLAIR) images with 3–4mm slice thickness and no interslice gap, and diffusion-weighted images with *b=*0, 500, and 1000 s/mm^2^, 3–4mm slice thickness and no interslice gap. Disease progression was determined using the modified RANO criteria^51^. Additionally, post-hoc quantitative tumor volumetric analysis was performed using contrast-enhanced T1-weighted digital subtraction maps and segmentation techniques described previously ^52–54^. Briefly, linear registration was first performed between all images including contrast enhanced T1-weighted images and T2-weighted and/or FLAIR images to nonenhanced T1-weighted images using a 12–degree-of-freedom transformation and a correlation coefficient cost function. Next, intensity normalization and bias field correction was performed for both nonenhanced and contrast enhanced T1-weighted images, and voxel-by-voxel subtraction between normalized nonenhanced and contrast-enhanced T1-weighted images was performed. Image voxels with a positive (greater than zero) before-to-after change in normalized contrast enhancement signal intensity (i.e., voxels increasing in MR signal after contrast agent administration) within T2-weighted FLAIR hyperintense regions were isolated to create the final T1 subtraction maps. Estimates of tumor volume included areas of contrast enhancement on T1 subtraction maps including central necrosis (defined as being enclosed by contiguous, positive enhancing disease).

### Patient samples.

Heparinized peripheral blood was collected at the baseline visit and at each treatment visit for immune monitoring. Peripheral blood mononuclear cells were collected in CPT tubes (BD Biosciences, cat: 362753), isolated according to the manufacturer’s protocol, placed in freezing media made of 90% human AB serum (Fisher Scientific, cat. MT35060CI) and 10% dimethyl sulfoxide (Sigma, cat. C6295–50ML) and stored in liquid nitrogen until the time of analysis. On the day of data acquisition, samples were thawed in a 37°C water bath and washed in RPMI-1640 media (Genesse Scientific, cat: 25–506) supplemented with FBS and penicillin and streptomycin. Patient tumor samples were attained immediately following surgery.

### Generation of autologous dendritic cell vaccines.

Monocyte-derived DCs were established from adherent peripheral blood mononuclear cells (PBMC) obtained via leukapheresis performed at the UCLA Hemapheresis Unit, as we have published on previously ^3,6,55^. All *ex vivo* DC preparations were performed in the UCLA-Jonsson Cancer Center GMP facility under sterile and monitored conditions. In brief, dendritic cells were prepared by culturing adherent cells from peripheral blood in RPMI-1640 (Gibco) and supplemented with 10% autologous serum, 500 U/mL GM-CSF (Leukine^®^, Amgen, Thousand Oaks, CA) and 500 U/mL of IL-4 (CellGenix), using techniques described previously ^56^. Following culture, DCs were collected by vigorous rinsing and washed with sterile 0.9% NaCl solution. The purity and phenotype of each DC lot was also determined by flow cytometry (FACScan flow cytometer; BD Biosciences, San Jose, CA). Cells were stained with FITC-conjugated CD83, PE-conjugated CD86 and PerCP-conjugated HLA-DR mAb’s (BD Biosciences). Release criteria were >70% viable by trypan blue exclusion, and >30% of the large cell gate being CD86^+^ and HLA-DR^+^. One day before each vaccination, DC were pulsed (co-cultured) with tumor lysate overnight, washed, and the final product was tested for sterility by Gram stain, mycoplasma and endotoxin testing prior to injection.

### Molecular and Immune Analyses

#### CyTOF.

Cells for mass cytometry analysis were prepared according to the Maxpar cell surface staining protocol. Briefly, 0.5 to 3 × 10^6^ cells were washed with PBS and treated with 0.1mg/mL of DNAse I Solution (StemCell Technologies, cat: 07900) for 15 minutes at room temperature. Cells were then resuspended in 5 μM Cell-ID cisplatin (Fluidigm, cat: 201064) as a live/dead marker for 5 minutes at room temperature. After quenching with the Maxpar cell staining buffer (Fluidigm, cat: 201068), the cells were incubated with a 23-marker panel for 30 minutes at room temperature. After washing, cells were incubated overnight in 125 nM iridium intercalation solution (1000X dilution of 125 μM Cell-ID Intercalator-Ir; Fluidigm, cat: 201192A) in Maxpar Fix and Perm Buffer (Fluidigm, cat: 201067) to label intracellular DNA. The next morning, cells were washed with cell staining buffer and distilled water. The samples were processed on a Helios mass cytometer (Fluidigm) in the University of California, Los Angeles Jonsson Comprehensive Cancer Center Flow Cytometry core.

The CyTOF data was normalized utilizing EQ four element calibration beads (Fluidigm, cat: 201078) with the R package *premessa* (version 0.2.4, Parker Institute for Cancer Immunotherapy) following removal of dead cells. A total of 5,000 cells were subsampled from each sample (except for sample S16-07-2-Day 1 where we only had 4,861 cells). Subsequently, bead normalized data from 45 samples were integrated as described previously^57^. Briefly, flow cytometry standard (FCS) files were loaded into R with the *flowCore* package (version 2.8.0). Raw marker intensities were transformed utilizing hyperbolic inverse sine (arcsinh) with cofactor of 5. Cell population identification was carried out using unsupervised clustering using *FlowSOM* package (version 2.4.0) and subsequent metaclustering using ConsensusClusterPlus package (version 1.60.0). The metaclusters were manually curated to identify canonical populations in Figure 2B (including one unknown cluster with little/no marker expression). The high dimensional data was visualized with the Uniform Manifold Approximation and Projection (UMAP). Differential marker analysis across treatment groups were first performed using the linear mixed model analysis pipeline as described^57^. Markers with nominally significant p-values in one or more cell populations (P ≤ 0.05; e.g CD39, CD38, Ki-67, PD-1) were visualized in boxplots; statistical significance computed using the linear mixed model were further confirmed using non-parametric Wilcoxon rank sum test.

#### Bulk RNAseq.

Total RNA was isolated from frozen PBMC of the patients isolated at baseline and after three biweekly vaccines with ATL-DC plus adjuvant (placebo: 5 pairs, resiquimod: 8 pairs and poly-ICLC: 8 pairs; see Supplementary Table Clinical) using the ZYMO quick RNA extraction kit. We utilized the TruSeq RNA exome kit to construct the RNA sequencing libraries. Paired-end, 2 × 100 base pair (bp) transcriptome reads were mapped to the Genome Reference Consortium Human Build 38 (GRCh38) reference genome using HISAT2 (version 2.0.6) ^58^. The gene level counts were generated by the HTSeq-count program (version 0.5.4p5) ^59^. We utilized the DESeq2 R package’s counts function (version 1.24.0)^60^ to compute the normalized gene expression values from the raw gene expression counts. DESeq2 normalized gene expression was log2 transformed after adding a pseudo count of 1. For subsequent differentially expressed genes (DEGs) and gene set enrichment analyses, we only included the known genes (i.e genes with RefSeq transcripts ID starting with “NM_”, that satisfy: 1) normalized expression IQR ≥ 1; and 2) normalized log2 expression ≥ 1 in at least one of the samples.

Based on the filtered gene list, we first obtained the patient-specific, log2 fold change of each gene before and after the ATL-DC vaccine treatment. Next, the mean of the log2 fold changes in the poly-ICLC or resiquimod group is compared to those in the placebo group. Genes showing at least 2-fold upregulation in any of the TLR agonist treated group (resiquimod or poly-ICLC, nominal t-test p-value ≤ 0.05) with respect to the placebo were tested for significant overlap with gene ontology and known gene sets using the web-based tools, ENRICHR ^61^.

To calculate single sample gene set enrichment of the interferon related genes, we used the Gene Set Variation Analysis (GSVA) package (version 1.32.0) ^62^. To compute the GSVA scores, the filtered, log2 normalized gene expression were supplied to the GSVA program using the ‘kcdf=Gaussian’ mode. We manually selected gene sets that are related to interferon pathway activation from the c2.cgp, c6, c7, hallmark geneset collections of the Broad Institute’s Molecular Signatures Database (version 7.0) ^63^.

#### Single-cell RNA-seq sample processing and data analysis.

The cells for scRNAseq analysis were resuspended in PBS at a concentration of 1,000 cells/μl. We only selected representative patients from each treatment group whose PBMC quality were sufficient for single cell RNAseq processing . Cell preparation, library preparation, and sequencing were carried out according to Chromium product-based manufacturer protocols (10X Genomics), targeting for a total of 10,000 cells sequenced. Single cell RNA sequencing was carried out on a Novaseq 6000 S2 2 × 50bp flow cell (Illumina) utilizing the Chromium single cell 3’ gene expression library preparation (10X Genomics).

The data was aligned with Cell Ranger (version 3.1.0) and aligned to the Genome Reference Consortium Human Build 38 (GRCh38). Data was imported into R (version 4.2.1) and analyzed with the Seurat package (version 4.2.0)^64^. For quality assurance, cells with greater than 20% mitochondrial features were excluded from further analysis. We analyzed a total of 99,590 cells after the QC step. The Seurat data object from each sample were then integrated and scaled, regressing out the percent mitochondrial features and cell cycle score difference, as described(https://satijalab.org/seurat/index.html). We manually identified each cluster using the genes that were differentially expressed as determined by *FindAllMarker* function; they are visualized using R’s ggplot2 and pheatmap packages. Differentially expressed genes (DEGs) corresponding to each treatment group (Placebo vs. Poly-ICLC vs. resiquimod) were computed by first computing cluster-specific DEGs between each group against the pre-treatment (Day 0) samples. The union of cluster-specific DEGs that were seen in at least 20% of all comparisons (the total number of comparisons is the number of treatment groups (3 groups) times number of lymphoid or myeloid clusters) were selected as recurrent DEGs shown in the heatmaps of Figure 2F and 2G.

### Statistical analysis.

For the percentage comparisons in the CyTOF analysis, we used the Wilcoxon rank sum test for non-parametric data for 2 independent samples and compared baseline (Day 0) to Day 1 or Day 29. We performed Fisher’s exact test for testing the null of independence of the phenotypic and genotypic characteristics and treatments using the *stats* package in R. Differences in transcript expression log2 fold changes FC and GSVA scores in the bulk RNA-seq data were calculated with unpaired T test with non-equal variances (two-sided Welch t test). The differences in overall survival or time to progression following treatment (either combination of ATL-DC and placebo, ATL-DC and adjuvant poly-ICLC or ATL-DC and adjuvant resiquimod treatment) were assessed using log-rank test (visualized using R’s survminer package). We further performed multivariable cox proportional hazard (cox PH) regression analysis with HRs (95% CIs) to determine if any of the treatment regimen were significantly predictive of overall survival or time to progression after adjusting for clinical covariates, such as WHO grade, number of recurrences, and MGMT status. The association between interferon pathway score and overall survival or time to progression was analyzed similarly using log-rank (univariate) and Cox PH (multivariate) analyses.

## Supplementary Material

Supplement 1

## Figures and Tables

**Table 1.** Baseline patient characteristics.

Variable	DC vaccine + placebo (n = 5)	DC vaccine + poly-ICLC (n =9)	DC vaccine + resiquimod (n=9)	Total (n=23)
Age (year)				
Mean (SD)	56.50 (13.75)	44.09 (12.04)	43.73 (8.80)	46.65 (11.98)
Median (IQR)	48.06	40.15	43.46	45.33
Sex				
Female, n (%)	2 (40%)	5 (56%)	3 (33%)	10 (43%)
Male, n (%)	3 (60%)	4 (44%)	6 (67%)	13 (57%)
OS (months)				
Mean (SD)	19.51 (23.49)	54.06 (34.63)	28.05 (23.82)	36–39 (30–93)
Median (iQr)	7.70	52.50	16.73	24.47
TTP (months)				
Mean (SD)	5.06 (1.10)	44.64 (36.26)	10.96 (8.64)	22.86 (28.80)
Median (IQR)	5.13	31.43	8.10	8.10
WHO grade, n (%)				
III	1 (20%)	4 (44%)	3 (33%)	7 (30%)
IV	4 (80%)	5 (56%)	7 (78%)	16 (70%)
Recurrence, n (%)				
None	1 (20%)	5 (56%)	3 (33%)	9 (39%)
Recurrence	4 (80%)	4 (44%)	6 (67%)	14 (61%)
MGMT status, n (%)				
Methylated	1 (20%)	4 (44%)	3 (33%)	8 (35%)
Unmethylated	4 (80%)	5 (56%)	6 (67%)	15 (65%)
EGFR classification, n (%)				
Amplified	3 (60%)	2 (22%)	5 (56%)	10 (44%)
Not amplified	1 (20%)	5 (56%)	3 (33%)	9 (39%)
Unknown	1 (20%)	2 (22%)	1 (11%)	4 (17%)
IDH status, n (%)				
Mutant	1 (20%)	4 (44%)	3 (33%)	8 (35%)
Wild-type	4 (80%)	5 (56%)	6 (67%)	15 (65%)
Avastin treatment, n (%)				
Treated	4 (80%)	5 (56%)	6 (67%)	15 (65%)
Not treated	1 (20%)	4 (44%)	3 (33%)	8 (35%)

**Table 2.** Adverse events across treatment cohorts.

Variable	DC vaccine + placebo (n = 5)	DC vaccine + poly-ICLC (n =9)	DC vaccine + resiquimod (n=9)	Total (n=23)
Any	1 (20%)	9 (100%)	8 (89%)	18 (78%)
Rash	0	1 (11%)	8 (89%)	9 (39%)
Fever	0	5 (56%)	3 (33%)	8 (35%)
Fatigue	1 (20%)	2 (22%)	3 (33%)	6 (26%)
Flu-like symptoms	0	2 (22%)	0	2 (9%)
Nasal congestion	0	1 (11%)	0	1 (4%)
Nervous system	0	4 (44%)	2 (22%)	4 (17%)
Headache	0	3 (33%)	1 (11%)	4 (17%)
Seizure	0	0	1 (11%)	1 (4%)
Sensory paresthesias	0	1 (11%)	0	1 (4%)
Cognitive disturbances	0	1 (11%)	0	1 (4%)
Ear pain	0	1 (11%)	0	1 (4%)
Musculoskeletal	0	3 (33%)	2 (22%)	5 (22%)
Neck pain	0	0	1 (11%)	1 (4%)
Body aches	0	1 (11%)	0	1 (4%)
Myalgia	0	2 (22%)	1 (11%)	3 (13%)
Gastrointestinal	0	1 (11%)	1 (11%)	2 (9%)
Nausea	0	1 (11%)	1 (11%)	2 (9%)
Vomiting	0	0	1 (11%)	1 (4%)
Cardiovascular / blood	0	0	2 (22%)	2 (9%)
Presyncope	0	0	1 (11%)	1 (4%)
Neutropenia	0	0	1 (11%)	1 (4%)

## Data Availability

Single-cell RNA sequencing data is available at Gene Expression Omnibus under accession ID GSE237581. The CyTOF data is uploaded to flowRepository with accession ID FR-FCM-Z6LY.

## Abbreviations:

GBM: Glioblastoma
TLR: toll-like receptor
DC: dendritic cell

## References

[1] Roger Stupp M.E.H., Mason Warren P, van den Bent Martin J, Taphoorn Martin J B, Janzer Robert C, Ludwin Samuel K, Allgeier Anouk, Fisher Barbara, Belanger Karl, Hau Peter, Brandes Alba A, Gijtenbeek Johanna, Marosi Christine, Vecht Charles J, Mokhtari Karima, Wesseling Pieter, Villa Salvador, Eisenhauer Elizabeth, Gorlia Thierry, Weller Michael, Lacombe Denis, Gregory Cairncross J, Mirimanoff René-Olivier. Effects of radiotherapy with concomitant and adjuvant temozolomide versus radiotherapy alone on survival in glioblastoma in a randomised phase III study: 5-year analysis of the EORTC-NCIC trial. Lancet Oncol 10, 459–466 (2009). 19269895 10.1016/S1470-2045(09)70025-7

[2] Linda M. Liau R.M.P., Kiertscher Sylvia M., Odesa Sylvia K, Kremen Thomas J, Giovannone Adrian J., Lin Jia-Wei, Chute Dennis J., Mischel Paul S., Cloughesy Timothy F., and Roth Michael D. Dendritic Cell Vaccination in Glioblastoma Patients Induces Systemic and Intracranial T-cell Responses Modulated by the Local Central Nervous System Tumor Microenvironment. Clin Cancer Res 11, 5515–5525 (2005). 16061868 10.1158/1078-0432.CCR-05-0464

[3] Prins R.M., Gene expression profile correlates with T-cell infiltration and relative survival in glioblastoma patients vaccinated with dendritic cell immunotherapy. Clin Cancer Res 17, 1603–1615 (2011). 21135147 10.1158/1078-0432.CCR-10-2563 PMC3071163

[4] Okada H., Induction of CD8+ T-cell responses against novel glioma-associated antigen peptides and clinical activity by vaccinations with {alpha}-type 1 polarized dendritic cells and polyinosinic-polycytidylic acid stabilized by lysine and carboxymethylcellulose in patients with recurrent malignant glioma. J Clin Oncol 29, 330–336 (2011). 21149657 10.1200/JCO.2010.30.7744 PMC3056467

[5] Liau Linda M., First results on survival from a large Phase 3 clinical trial of an autologous dendritic cell vaccine in newly diagnosed glioblastoma. J Transl Med 16, 1–9 (2018). 29843811 10.1186/s12967-018-1507-6 PMC5975654

[6] Liau L.M., Association of Autologous Tumor Lysate-Loaded Dendritic Cell Vaccination With Extension of Survival Among Patients With Newly Diagnosed and Recurrent Glioblastoma: A Phase 3 Prospective Externally Controlled Cohort Trial. JAMA Oncol 9, 112–121 (2023). 36394838 10.1001/jamaoncol.2022.5370 PMC9673026

[7] Yu M. & Levine S.J. Toll-like receptor, RIG-I-like receptors and the NLRP3 inflammasome: key modulators of innate immune responses to double-stranded RNA viruses. Cytokine Growth Factor Rev 22, 63–72 (2011). 21466970 10.1016/j.cytogfr.2011.02.001 PMC3109132

[8] Medzhitov R. Recognition of microorganisms and activation of the immune response. Nature 449, 819–826 (2007). 17943118 10.1038/nature06246

[9] Huang B., Zhao J., Unkeless J.C., Feng Z.H. & Xiong H. TLR signaling by tumor and immune cells: a double-edged sword. Oncogene 27, 218–224 (2008). 18176603 10.1038/sj.onc.1210904

[10] Wang R.F., Miyahara Y. & Wang H.Y. Toll-like receptors and immune regulation: implications for cancer therapy. Oncogene 27, 181–189 (2008). 18176599 10.1038/sj.onc.1210906

[11] Kawasaki T. & Kawai T. Toll-like receptor signaling pathways. Front Immunol 5, 461 (2014). 25309543 10.3389/fimmu.2014.00461 PMC4174766

[12] Matsumoto M., Subcellular localization of Toll-like receptor 3 in human dendritic cells. J Immunol 171, 3154–3162 (2003). 12960343 10.4049/jimmunol.171.6.3154

[13] Seya T., Funami K., Taniguchi M. & Matsumoto M. Antibodies against human Toll-like receptors (TLRs): TLR distribution and localization in human dendritic cells. J Endotoxin Res 11, 369–374 (2005). 16303093 10.1179/096805105X67292

[14] McKimmie C.S. & Fazakerley J.K. In response to pathogens, glial cells dynamically and differentially regulate Toll-like receptor gene expression. J Neuroimmunol 169, 116–125 (2005). 16146656 10.1016/j.jneuroim.2005.08.006

[15] Olson J.K. & Miller S.D. Microglia initiate central nervous system innate and adaptive immune responses through multiple TLRs. J Immunol 173, 3916–3924 (2004). 15356140 10.4049/jimmunol.173.6.3916

[16] Carpentier P.A., Differential activation of astrocytes by innate and adaptive immune stimuli. Glia 49, 360–374 (2005). 15538753 10.1002/glia.20117

[17] Farina C., Preferential expression and function of Toll-like receptor 3 in human astrocytes. J Neuroimmunol 159, 12–19 (2005). 15652398 10.1016/j.jneuroim.2004.09.009

[18] Bsibsi M., Toll-like receptor 3 on adult human astrocytes triggers production of neuroprotective mediators. Glia 53, 688–695 (2006). 16482523 10.1002/glia.20328

[19] Zhu X., Toll like receptor-3 ligand poly-ICLC promotes the efficacy of peripheral vaccinations with tumor antigen-derived peptide epitopes in murine CNS tumor models. J Transl Med 5, 10 (2007). 17295916 10.1186/1479-5876-5-10 PMC1802742

[20] Guarda G., Type I interferon inhibits interleukin-1 production and inflammasome activation. Immunity 34, 213–223 (2011). 21349431 10.1016/j.immuni.2011.02.006

[21] Sultan H., Wu J., Kumai T., Salazar A.M. & Celis E. Role of MDA5 and interferon-I in dendritic cells for T cell expansion by anti-tumor peptide vaccines in mice. Cancer Immunol Immunother 67, 1091–1103 (2018). 29696308 10.1007/s00262-018-2164-6 PMC6585404

[22] Zhu X., Poly-ICLC promotes the infiltration of effector T cells into intracranial gliomas via induction of CXCL10 in IFN-alpha and IFN-gamma dependent manners. Cancer Immunol Immunother 59, 1401–1409 (2010). 20549206 10.1007/s00262-010-0876-3 PMC2893267

[23] Kyi C., Therapeutic Immune Modulation against Solid Cancers with Intratumoral Poly-ICLC: A Pilot Trial. Clin Cancer Res 24, 4937–4948 (2018). 29950349 10.1158/1078-0432.CCR-17-1866 PMC6191332

[24] Giantonio B.J., Toxicity and response evaluation of the interferon inducer poly ICLC administered at low dose in advanced renal carcinoma and relapsed or refractory lymphoma: a report of two clinical trials of the Eastern Cooperative Oncology Group. Invest New Drugs 19, 89–92 (2001). 11291838 10.1023/a:1006458232384

[25] Salazar A.M., Long-term treatment of malignant gliomas with intramuscularly administered polyinosinic-polycytidylic acid stabilized with polylysine and carboxymethylcellulose: an open pilot study. Neurosurgery 38, 1096–1103; discussion 1103–1094 (1996). 8727138

[26] Butowski N., A phase II clinical trial of poly-ICLC with radiation for adult patients with newly diagnosed supratentorial glioblastoma: a North American Brain Tumor Consortium (NABTC01–05). J Neurooncol 91, 175–182 (2009). 18797818 10.1007/s11060-008-9693-3 PMC4779120

[27] Smits E.L., Ponsaerts P., Berneman Z.N. & Van Tendeloo V.F. The use of TLR7 and TLR8 ligands for the enhancement of cancer immunotherapy. Oncologist 13, 859–875 (2008). 18701762 10.1634/theoncologist.2008-0097

[28] Prins R.M., The TLR-7 agonist, imiquimod, enhances dendritic cell survival and promotes tumor antigen-specific T cell priming: relation to central nervous system antitumor immunity. J Immunol 176, 157–164 (2006). 16365406 10.4049/jimmunol.176.1.157

[29] Chang B.A., Cross J.L., Najar H.M. & Dutz J.P. Topical resiquimod promotes priming of CTL to parenteral antigens. Vaccine 27, 5791–5799 (2009). 19660592 10.1016/j.vaccine.2009.07.062

[30] Du J., TLR8 agonists stimulate newly recruited monocyte-derived cells into potent APCs that enhance HBsAg immunogenicity. Vaccine 28, 6273–6281 (2010). 20637759 10.1016/j.vaccine.2010.06.117 PMC3031106

[31] Rajagopal D., Plasmacytoid dendritic cell-derived type I interferon is crucial for the adjuvant activity of Toll-like receptor 7 agonists. Blood 115, 1949–1957 (2010). 20065291 10.1182/blood-2009-08-238543 PMC2837337

[32] Nair S., Injection of immature dendritic cells into adjuvant-treated skin obviates the need for ex vivo maturation. J Immunol 171, 6275–6282 (2003). 14634145 10.4049/jimmunol.171.11.6275

[33] Canale F.P., CD39 Expression Defines Cell Exhaustion in Tumor-Infiltrating CD8+ T Cells. Cancer Research 78, 115–128 (2018). 29066514 10.1158/0008-5472.CAN-16-2684

[34] Philip M., Chromatin states define tumour-specific T cell dysfunction and reprogramming. Nature 545, 452–456 (2017). 28514453 10.1038/nature22367 PMC5693219

[35] Vignali P.D.A., Hypoxia drives CD39-dependent suppressor function in exhausted T cells to limit antitumor immunity. Nature Immunology 24, 267–279 (2023). 36543958 10.1038/s41590-022-01379-9 PMC10402660

[36] Swaim C.D., Scott A.F., Canadeo L.A. & Huibregtse J.M. Extracellular ISG15 Signals Cytokine Secretion through the LFA-1 Integrin Receptor. Mol Cell 68, 581–590 e585 (2017). 29100055 10.1016/j.molcel.2017.10.003 PMC5690536

[37] Zhang X., Human intracellular ISG15 prevents interferon-alpha/beta over-amplification and auto-inflammation. Nature 517, 89–93 (2015). 25307056 10.1038/nature13801 PMC4303590

[38] Gough D.J., Functional crosstalk between type I and II interferon through the regulated expression of STAT1. PLoS Biol 8, e1000361 (2010). 20436908 10.1371/journal.pbio.1000361 PMC2860501

[39] Zhang Y., PARP9-DTX3L ubiquitin ligase targets host histone H2BJ and viral 3C protease to enhance interferon signaling and control viral infection. Nat Immunol 16, 1215–1227 (2015). 26479788 10.1038/ni.3279 PMC4653074

[40] Park H., Polyinosinic-polycytidylic acid is the most effective TLR adjuvant for SIV Gag protein-induced T cell responses in nonhuman primates. J Immunol 190, 4103–4115 (2013). 23509365 10.4049/jimmunol.1202958 PMC3622154

[41] Saxena M., Poly-ICLC, a TLR3 Agonist, Induces Transient Innate Immune Responses in Patients With Treated HIV-Infection: A Randomized Double-Blinded Placebo Controlled Trial. Front Immunol 10, 725 (2019). 31024557 10.3389/fimmu.2019.00725 PMC6467168

[42] Sultan H., Poly-IC enhances the effectiveness of cancer immunotherapy by promoting T cell tumor infiltration. J Immunother Cancer 8(2020). 10.1136/jitc-2020-001224 PMC7507896 32958686

[43] Ott M., Prins R.M. & Heimberger A.B. The immune landscape of common CNS malignancies: implications for immunotherapy. Nat Rev Clin Oncol 18, 729–744 (2021). 34117475 10.1038/s41571-021-00518-9 PMC11090136

[44] Jiang W., Exhausted CD8+T Cells in the Tumor Immune Microenvironment: New Pathways to Therapy. Front Immunol 11, 622509 (2020). 33633741 10.3389/fimmu.2020.622509 PMC7902023

[45] Reardon D.A., Effect of Nivolumab vs Bevacizumab in Patients With Recurrent Glioblastoma: The CheckMate 143 Phase 3 Randomized Clinical Trial. JAMA Oncol 6, 1003–1010 (2020). 32437507 10.1001/jamaoncol.2020.1024 PMC7243167

[46] Hiam-Galvez K.J., Allen B.M. & Spitzer M.H. Systemic immunity in cancer. Nat Rev Cancer 21, 345–359 (2021). 33837297 10.1038/s41568-021-00347-z PMC8034277

[47] Marshall A.S.J., Human MICL (CLEC12A) is differentially glycosylated and is downregulated following cellular activation. European Journal of Immunology 36, 2159–2169 (2006). 16838277 10.1002/eji.200535628

[48] Neumann K., Clec12a is an inhibitory receptor for uric acid crystals that regulates inflammation in response to cell death. Immunity 40, 389–399 (2014). 24631154 10.1016/j.immuni.2013.12.015

[49] Krieg C., High-dimensional single-cell analysis predicts response to anti-PD-1 immunotherapy. Nature Medicine 24, 144–153 (2018). 10.1038/nm.4466 29309059

[50] Ellingson B.M., Consensus recommendations for a standardized Brain Tumor Imaging Protocol in clinical trials. Neuro Oncol 17, 1188–1198 (2015). 26250565 10.1093/neuonc/nov095 PMC4588759

[51] Wen P.Y., Updated response assessment criteria for high-grade gliomas: response assessment in neuro-oncology working group. J Clin Oncol 28, 1963–1972 (2010). 20231676 10.1200/JCO.2009.26.3541

[52] Ellingson B.M., Volumetric response quantified using T1 subtraction predicts long-term survival benefit from cabozantinib monotherapy in recurrent glioblastoma. Neuro Oncol 20, 1411–1418 (2018). 29660005 10.1093/neuonc/noy054 PMC6120362

[53] Ellingson B.M., Recurrent glioblastoma treated with bevacizumab: contrast-enhanced T1-weighted subtraction maps improve tumor delineation and aid prediction of survival in a multicenter clinical trial. Radiology 271, 200–210 (2014). 24475840 10.1148/radiol.13131305 PMC4263651

[54] Ellingson B.M., Contrast-enhancing tumor growth dynamics of preoperative, treatment-naive human glioblastoma. Cancer 122, 1718–1727 (2016). 26998740 10.1002/cncr.29957

[55] Prins R.M., Cloughesy T.F. & Liau L.M. Cytomegalovirus Immunity after Vaccination with Autologous Glioblastoma Lysate. New England Journal of Medicine 359, 539–541 (2008). 18669440 10.1056/NEJMc0804818 PMC2775501

[56] Liau L.M., Dendritic cell vaccination in glioblastoma patients induces systemic and intracranial T-cell responses modulated by the local central nervous system tumor microenvironment. Clin Cancer Res 11, 5515–5525 (2005). 16061868 10.1158/1078-0432.CCR-05-0464

[57] Nowicka M., CyTOF workflow: differential discovery in high-throughput high-dimensional cytometry datasets. F1000Res 6, 748 (2017). 28663787 10.12688/f1000research.11622.1 PMC5473464

[58] Kim D., Langmead B. & Salzberg S.L. HISAT: a fast spliced aligner with low memory requirements. Nat Methods 12, 357–360 (2015). 25751142 10.1038/nmeth.3317 PMC4655817

[59] Anders S., Pyl P.T. & Huber W. HTSeq--a Python framework to work with high-throughput sequencing data. Bioinformatics 31, 166–169 (2015). 25260700 10.1093/bioinformatics/btu638 PMC4287950

[60] Love M.I., Huber W. & Anders S. Moderated estimation of fold change and dispersion for RNA-seq data with DESeq2. Genome Biol 15, 550 (2014). 25516281 10.1186/s13059-014-0550-8 PMC4302049

[61] Kuleshov M.V., Enrichr: a comprehensive gene set enrichment analysis web server 2016 update. Nucleic Acids Res 44, W90–97 (2016). 27141961 10.1093/nar/gkw377 PMC4987924

[62] Hanzelmann S., Castelo R. & Guinney J. GSVA: gene set variation analysis for microarray and RNA-seq data. BMC Bioinformatics 14, 7 (2013). 23323831 10.1186/1471-2105-14-7 PMC3618321

[63] Subramanian A., Gene set enrichment analysis: a knowledge-based approach for interpreting genome-wide expression profiles. Proc Natl Acad Sci U S A 102, 15545–15550 (2005). 16199517 10.1073/pnas.0506580102 PMC1239896

[64] Butler A., Hoffman P., Smibert P., Papalexi E. & Satija R. Integrating single-cell transcriptomic data across different conditions, technologies, and species. Nat Biotechnol 36, 411–420 (2018). 29608179 10.1038/nbt.4096 PMC6700744

