# Supplementary material for: Dendritic Cell Vaccination in Conjunction with a TLR Agonist Polarizes Interferon Immune Responses in Malignant Glioma Patients
Source: Res Sq. 2023 Sep 12:rs.3.rs-3287211. Preprint. [Version 1] doi: 10.21203/rs.3.rs-3287211/v1 (PMC10543402; doi:10.21203/rs.3.rs-3287211/v1)
Supplement: Supplement 1 [file NIHPPrs3287211v1-supplement-1.pdf]

Supplementary Table 1. Patient level clinical characteristics.

Supplementary Table 2. Patient-level gene expression and gene set scores from bulk RNA-seq data.

A, Difference in log fold changes in ATL-DC+TLR agonist groups compared to ATL-DC+placebo.

B, Gene sets within ENRICHR database that are significantly overlapping with DEGs upregulated in TLR agonist-treated groups (poly-ICLC or resiquimod, average log2 FC ≥ 1, nominal P-value ≤ 0.05).

C, Patient-level gene expression changes in pre- vs. post-treatment samples (log fold changes). This is the raw data for the gene expression boxplots.
D, Patient-level GSVA gene set score change in pre- vs. post-treatment samples (computed over c2 cgp, c6, c7 and hallmark subsets of MSigDB). This is the raw data for the GSVA score boxplots.
E, Patient-level GSVA score of interferon related gene sets in pre- vs. post-treatment samples. This is the raw data of the GSVA heatmap.

Supplementary Table 3. Cell population identification in CyTOF and single cell RNAseq datasets.
A, CyTOF sample characteristics.
B, CyTOF marker list.
C, Differentially expressed CyTOF markers computed by linear mixed model analysis.
D, Sample-level fractions of cell types identified by unsupervised clustering of the CyTOF dataset.
E, Single cell RNAseq sample characteristics.
F, Cell types identified by cluster-specific, differentially expressed transcripts in the single cell RNAseq dataset.
G, Differentially expressed genes in the post-treatment samples of each treatment group (placebo, poly-ICLC and resiquimod) with respect to all pre-treatment samples. This is the raw data of the single cell RNAseq heatmap.

Supplementary Table 4. Univariate and multivariate analyses testing the association between IFN gene set scores and patient survival.
A, The association between progression free survival (PFS) or overall survival (OS) and the GSVA scores of select type I and type II interferon gene sets (univariate analysis, P values, log-rank; preTx=GSVA score of pre-treatment sample, postTx=GSVA score of post-treatment sample, diff_preTx_postTx=postTx-preTx).
B, Cox proportional hazard analysis testing the association between PFS or OS and the GSVA scores of select type I and type II interferon gene sets.

Supplementary Figure 1. CONSORT diagram of clinical trial enrollment.

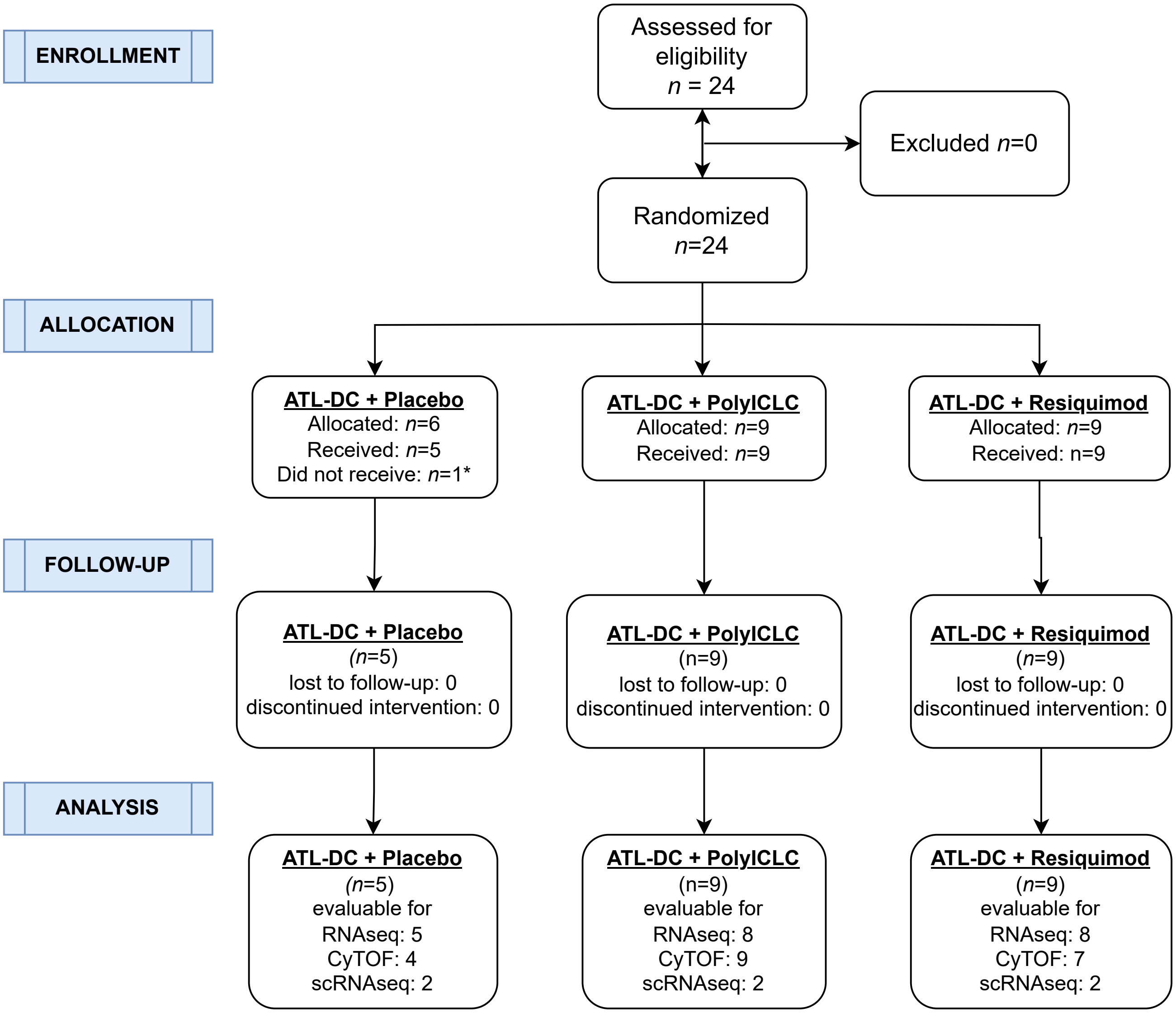

*due to early progression during vaccine manufacture

Supplementary Figure 2

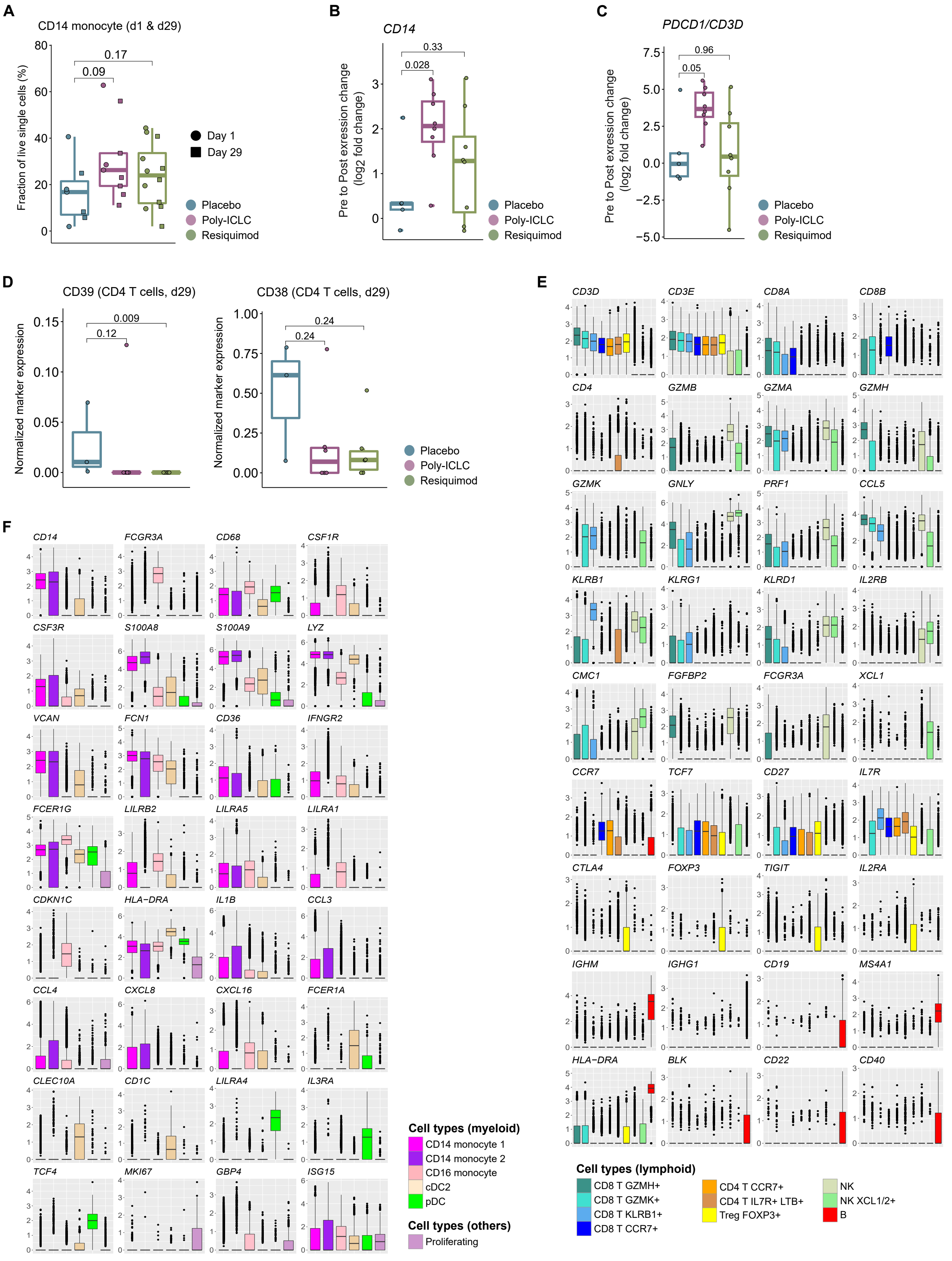

Supplementary Figure 2. CyTOF and single cell transcriptomics of patient PBMCs before and after ATL-DC vaccine with or without adjuvant TLR agonist.
A, Comparison of CD14+ monocyte fraction in post-treatment PBMCs of patients from indicated treatment groups. P values, two-sided Wilcoxon rank sum test.
B, Differential gene expression (pre vs. post-treatment fold change, in $\log_2$) of *CD14* transcript across treatment groups (P values, two-sided two-sided Welch t test).
C, Differential gene expression (pre vs. post-treatment fold change, in $\log_2$) of *PDCD1* transcript across treatment groups (P values, two-sided two-sided Welch t test) after adjusting for the change in *CD3D* transcript expression in the same sample pair. The values approximate the changes of *PDCD1* transcript per T cell.
D, Normalized expression of indicated markers in CD4 T cell populations within the PBMC samples of patients from indicated treatment groups. P values, two-sided Wilcoxon rank sum test.
E, F, Boxplots showing marker gene expressions in lymphoid cell populations (E) or myeloid and proliferative cell populations (F).

Supplementary Figure 3

A

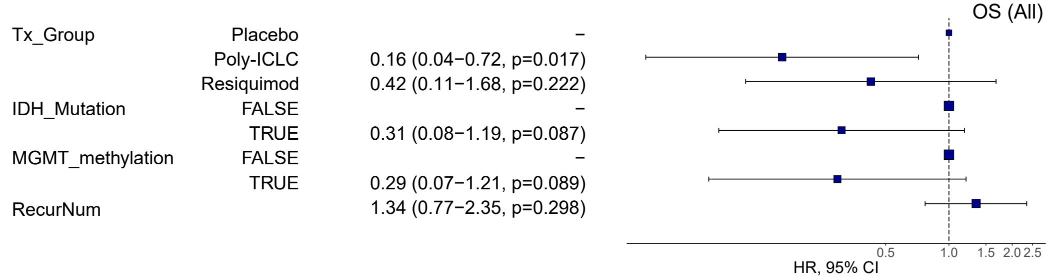

B

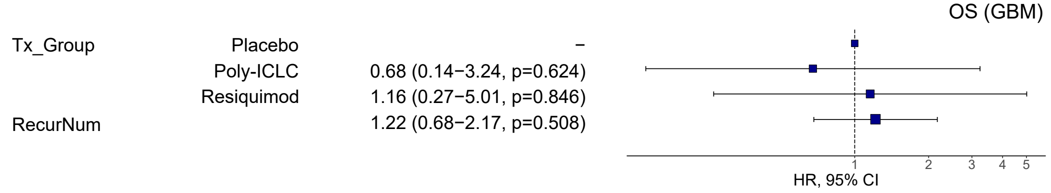

C

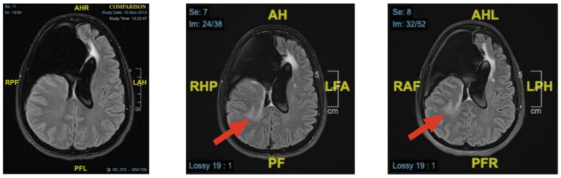

Pre-vaccination (Nov 2013)

Post-vaccination

Post-vaccination (Jun 2022, 9 years)

D

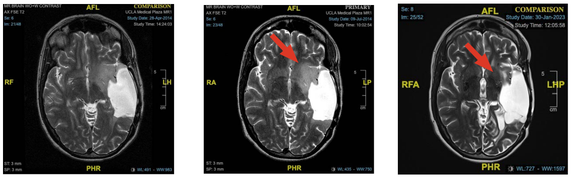

Pre-vaccination (Apr 2014)

Post-vaccination

Post-vaccination (Jan 2023, 9 years)

Supplementary Figure 3. The association between combined ATL-DC vaccine and TLR agonist and patient survival.

A, B, Multivariate Cox proportional hazards analysis assessing the hazard ratios of death in TLR agonist treatment groups against placebo in all patients (A) or GBM subset (B) after adjusting for other clinical covariates (Tx_Group=treatment group, RecurNum=number of recurrences prior to ATL-DC treatment; the CoxPH model did not converge when MGMT_methylation was included).

C, D, Representative contrast-enhanced MR imaging of patients treated with ATL-DC + poly-ICLC showing initial increase of T2/FLAIR MRI signal (red arrows), which either persists (G) or regresses (H) over time. Both patients have significantly longer PFS and OS than the rest of the patients in the cohort.

Supplementary Figure 4

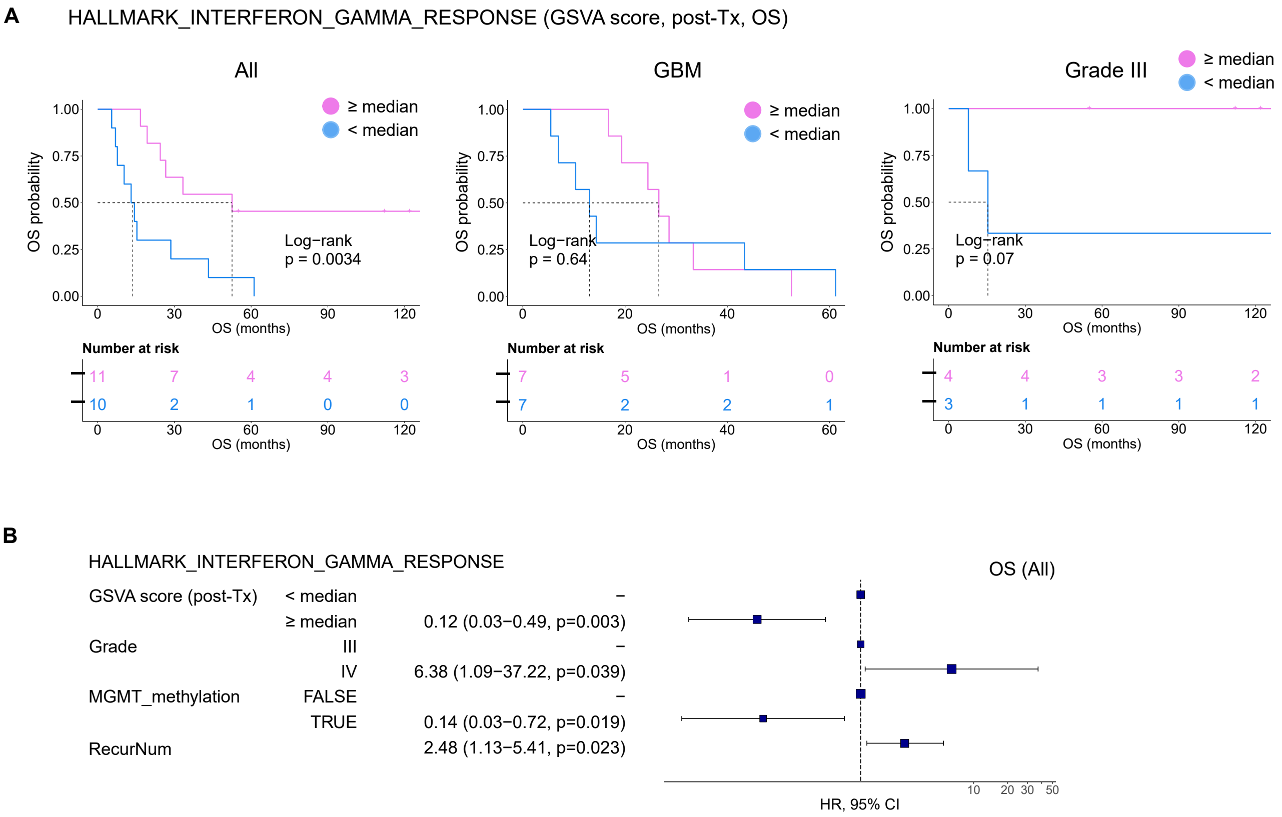

Supplementary Figure 4. The association between IFN pathway activation and overall survival after ATL-DC vaccine and TLR agonist therapy.
A, Kaplan-Meier overall survival curves of all patients (left), GBM (center), and Grade III glioma subsets (right) stratified by their HALLMARK_INTERFERON_GAMMA_RESPONSE GSVA scores in their post-treatment PBMCs. P values, log-rank test.
B, Multivariate Cox proportional hazards analysis assessing hazard ratios of death in patients with high HALLMARK_INTERFERON_GAMMA_RESPONSE GSVA score after adjusting for other clinical covariates.
